# A Glycosphingolipid Binding Domain Controls Trafficking and Activity of the Mammalian Notch Ligand Delta-Like 1

**DOI:** 10.1371/journal.pone.0074392

**Published:** 2013-09-12

**Authors:** Sara Farrah Heuss, Nadine Tarantino, Jacques Fantini, Delphine Ndiaye-Lobry, Julien Moretti, Alain Israël, Frédérique Logeat

**Affiliations:** 1 Unité de Signalisation Moléculaire et Activation Cellulaire, URA CNRS 2582, Institut Pasteur, Paris, France; 2 Laboratoire des Interactions Moléculaires et Systèmes Membranaires, UMR CNRS 6231, Faculté des Sciences de Saint Jérôme, Université d’Aix-Marseille, Marseille, France; J. Heyrovsky Institute of Physical Chemistry, Czech Republic

## Abstract

The activity of Notch ligands is tightly regulated by trafficking events occurring both before and after ligand-receptor interaction. In particular endocytosis and recycling have been shown to be required for full signaling activity of the ligands before they encounter the Notch receptor. However little is known about the precise endocytic processes that contribute to ligand internalization. Here we demonstrate that endocytosis contributes to Dll1 signaling activity by preserving the ligand from shedding and degradation. We further show that the glycosphingolipid-binding motif originally identified in Drosophila Notch ligands is conserved in mammals and is necessary for Dll1 internalization. Mutation of its conserved tryptophan residue results in a Dll1 molecule which is rapidly inactivated by shedding and degradation, does not recycle to the cell surface and does not activate Notch signaling. Finally, silencing in the signal-sending cells of glucosylceramide synthase, the enzyme implicated in the initial phase of glycosphingolipid synthesis, down-regulates Notch activation. Our data indicate that glycosphingolipids, by interacting with Dll1, may act as functional co-factors to promote its biological activity.

## Introduction

Notch signaling is an evolutionary-conserved pathway involved in cell-cell communication [1]. At the cell surface, Notch receptors are present as heterodimers [2,3,4] consisting of a large N-terminal extracellular domain non-covalently bound to a C-terminal membrane- anchored domain. Upon interaction with a Delta/Serrate/Lag-2 (DSL) ligand, Notch receptors undergo two proteolytic cleavages that lead to transcriptional activation of Notch target genes. Despite the apparent simplicity of this pathway, Notch activation is tightly regulated at multiple levels, both in the signal-emitting and signal-receiving cell [5,6]. Endocytosis and endosomal trafficking have been shown to play an important role in the activation and regulation of Notch signaling [7]. In particular, several studies have pointed to the importance of endocytosis and recycling of the ligand in signal-emitting cells [8,9]. However the precise mechanism by which ligand endocytosis and recycling contribute to Notch activation remains debated [10]. Two possible non-exclusive models have been proposed to explain how ligand endocytosis could activate Notch signaling: i) prior to Notch binding, endocytosis and recycling would be required to generate an active surface-expressed ligand, and/or to maintain a certain level of ligand at the cell surface, ii) following interaction with the receptor, endocytosis of the ligand in the signal-sending cell would produce a mechanical force sufficient to induce structural changes in the receptor, allowing its proteolytic cleavage and subsequent activation of the pathway [11].

These 2 types of endocytic events might be mutually exclusive, or occur consecutively, the first one being required to “activate” the ligand, the second one to allow “pulling” and thus activation of the Notch receptor [12]. Several studies suggest that DSL ligands have to be internalized through clathrin-mediated endocytosis to become active [11,13,14]. However this requirement is highly context-dependent, e.g. clathrin is dispensable in the signal-sending cell for Notch activation in the Drosophila ovary [15]. A number of endocytic proteins required in signal-sending cells for ligand endocytosis and signaling have been identified, including dynamin, auxilin, epsin, Rab11 (but see 16,17), CALM, but the precise function of these proteins is still debated and may differ in specific developmental contexts [16,17,18].

In addition to clathrin-mediated endocytosis, cell surface proteins can be internalized through several types of non-clathrin endocytosis pathways [19]. Some of these pathways rely on the existence of membrane subdomains enriched in cholesterol- and sphingolipids [20]. These domains have been first characterized by their resistance to detergent solubilization, and while their existence was originally debated, recent microscopic and spectroscopic approaches support their existence in living cells [21]. The involvement of these domains in Notch signaling has been proposed in the case of the formation of sensory organ precursors in Drosophila [22,23,24,25]. We have previously shown that the Notch ligand Delta-like1 (Dll1) essentially localizes to these detergent-resistant membranes (DRMs), contrary to non-active mutants [26], suggesting that these domains are involved in the regulation of Dll1 signaling activity. The function of this microenvironment could be to select and concentrate molecules in order to facilitate signaling and/or to participate in ligand trafficking. Many receptors, like the EGF receptor, are known to undergo both clathrin- dependent and -independent endocytosis [27], and it has been reported that segregation of the EGF receptor, the TGFβ receptor and LRP6 into distinct membrane compartments determines their fate, i.e. degradation or recycling [5]. Hamel and collaborators have demonstrated in Drosophila that the composition of the plasma membrane can modulate ligand endocytosis and signaling activity and have identified in the extracellular domain of Drosophila Delta a structural motif (glycosphingolipid-binding motif or GBM) known to trigger interaction with glycosphingolipids [28], but the role of this motif has not been addressed directly. Glycosphingolipids anchored in the outer leaflet of the cellular plasma membrane are frequently associated with sphingomyelin and cholesterol to promote the formation of membrane subdomains. Glycosphingolipids play important roles in a variety of cellular events including differentiation, adhesion, growth and protein trafficking [29,30]. Interestingly, in *Caenorhabditis elegans* genetic studies have identified BRE-5 (the homologue of Drosophila Brainiac), a glycosyltransferase involved in glycosphingolipid biosynthesis, as a non-cell-autonomous regulator of Notch signaling, raising the possibility that glycosphingolipids could modulate the signaling activity of Notch ligands [28,31]. The purpose of our study was to investigate in more details the role of the lipid composition of the plasma membrane (and hence of specific subdomains) in Dll1 trafficking, and more specifically the existence of a glycosphingolipid-binding motif in mammalian Dll1 and its potential role in Dll1 trafficking and activity. We demonstrate here that a GBM exists in Dll1, and that it is required for proper trafficking of Dll1 by allowing protection from degradation and shedding, and ultimately for activation of the pathway. The first step in glycosphingolipid synthesis is catalyzed by glucosylceramide synthase (GCS), a limiting enzyme controlling the intracellular level of more than 300 species of glycosphingolipids [32]. The present study shows that down-regulation of GCS by shRNA in signal-sending cells inhibits Dll1-dependent Notch activation.

Our results indicate that altering the lipid composition of the plasma membrane and the ability of Dll1 to interact with these lipids has profound effects on ligand trafficking and signaling activity.

## Results

### The lipid composition of the plasma membrane regulates Dll1 shedding and turnover

We have previously reported that wild type (wt) Dll1 can be detected in fractions containing detergent-resistant membranes (DRMs) after flotation in a sucrose gradient, while non-active mutants of Dll1 do not localize to these fractions [26]. In order to determine the importance of the lipid composition of the plasma membrane, and thus of its potential subdomain organization, we interfered with this organization by treating cells with cholesterol oxidase (coase, leading to conversion of cholesterol to cholestenone) and sphingomyelinase (smase, leading to hydrolysis of sphingomyelin) [33,34]. We then determined whether this treatment affects Dll1 distribution in a sucrose gradient of cell extracts prepared with 1% Brij98.

**Figure 1:**
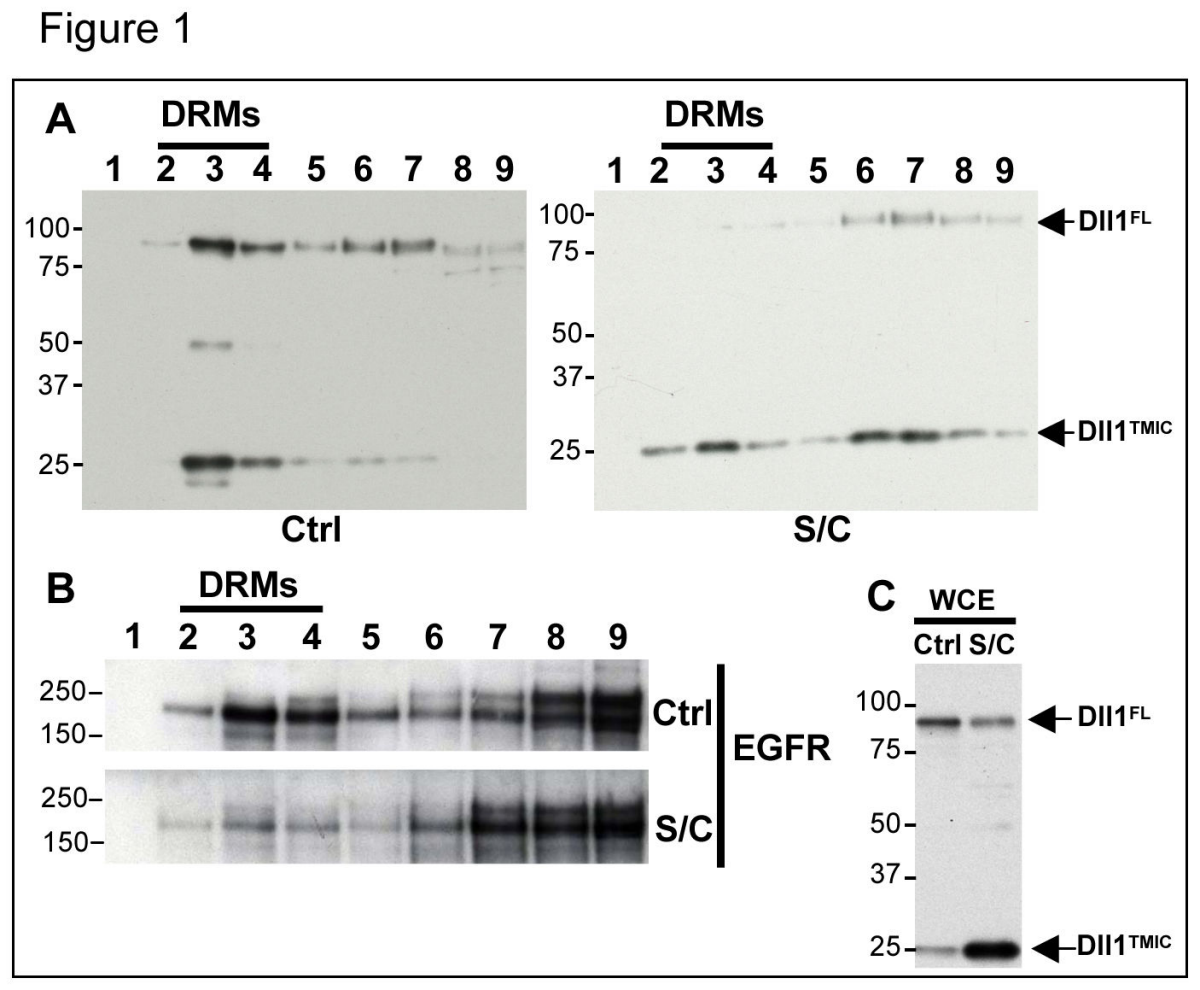
Cell treatment with smase/coase affects the localization of Dll1 in a sucrose gradient. VSV-Dll1-expressing OP9 cells, treated with 0.1 unit/mL sphingomyelinase and 1 unit/mL cholesterol oxidase (S/C) or not (Ctrl), were lysed with 1% Brij98 and lysates were subjected to fractionation on sucrose density gradients. Fractions were collected from the top (fraction 1, low density) to the bottom (fraction 9, high density) and analyzed by immunoblotting with anti-Dll1 (A) and anti-EGF receptor (EGFR) (B) antibodies. DRMs: Detergent Resistant Membranes. (C) Western blot analysis of whole cell extracts (WCE) with anti-Dll1 antibody. Molecular mass markers (kDa) are indicated on the left. Dll1FL: full-length form of the ligand. Dll1TMIC: membrane associated metalloprotease cleavage product.

**Figure 2:**
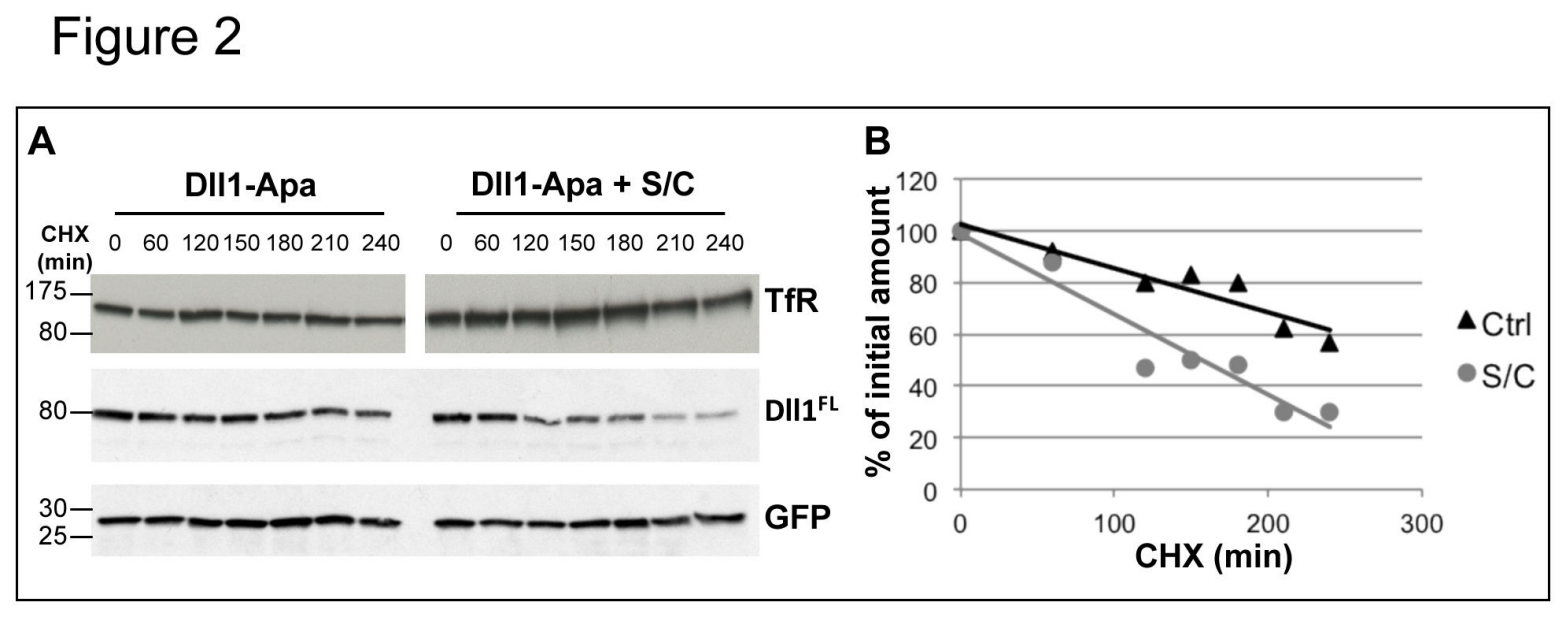
Cell treatment with smase/coase increases Dll1 turnover. HeLa cells were transiently-transfected with plasmids encoding Dll1-Apa and GFP. Cells were incubated in the absence or presence of 0.1 unit/mL sphingomyelinase and 1 unit/mL cholesterol oxidase (S/C) and lysed at the indicated time (min) following cycloheximide treatment. Dll1FL, GFP and endogenous transferrin receptor (TfR) were detected by western blotting. A graphic representation of the relative abundance of Dll1, quantified using the Quantity One software (Biorad), is shown in the bottom panel. This result is representative of 3 independent experiments.

Treatment of cells with smase/coase led to complete redistribution of full-length Dll1 into heavier fractions (Figure 1A). As a control, the EGF receptor, which has been demonstrated to be associated with DRMs [35], was found to be also redistributed to detergent-soluble fractions (Figure 1B, fractions 7 to 9); on the other hand, the transferrin receptor (TfR) was found to be associated with the heavy fractions, and this localization was not affected by smase/coase treatment (not shown). The TMIC fragment produced by metalloproteinase cleavage of Dll1 was also found associated with DRMs fractions, and redistributed to soluble fractions (although partially) after smase/coase treatment. We also observed that smase/coase treatment increases the ratio TMIC/FL (Figure 1C; this explains the apparent unequal protein loading seen in sucrose gradient), probably because of increased metalloproteinase-mediated shedding, associated with the high stability of the TMIC fragment (not shown). This phenomenon can be explained if smase/coase treatment inhibits Dll1 endocytosis and increases the amount of ligand at the cell surface, where it is more likely to undergo metalloprotease cleavage [36]. Next, we measured the levels of Dll1 in cells treated or not with smase/coase following cycloheximide treatment. Since smase/coase treatment was shown in Figure 1 to modulate Dll1 cleavage, in order to accurately measure the stability of the full-length molecule we took advantage of a mutant of Dll1 that we previously generated (Dll1-Apa [36]), which is resistant to shedding by membrane metalloproteases of the ADAM family. We transiently-transfected HeLa cells with Dll1-Apa and GFP. As shown in Figure 2, the half-life of Dll1 is clearly shorter in smase/coase treated cells. By contrast the stability of the transferrin receptor and that of GFP were not affected. These findings indicate that full-length Dll1 is more accessible to degradation when lipid organization of the plasma membrane is disrupted. One possible explanation for this increased degradation rate is that association of Dll1 with certain membrane lipids allows the ligand to escape lysosomal degradation by being internalized and redirected to recycling endosomes.

### Dll1 trafficking and membrane compartmentalization

**Figure 3:**
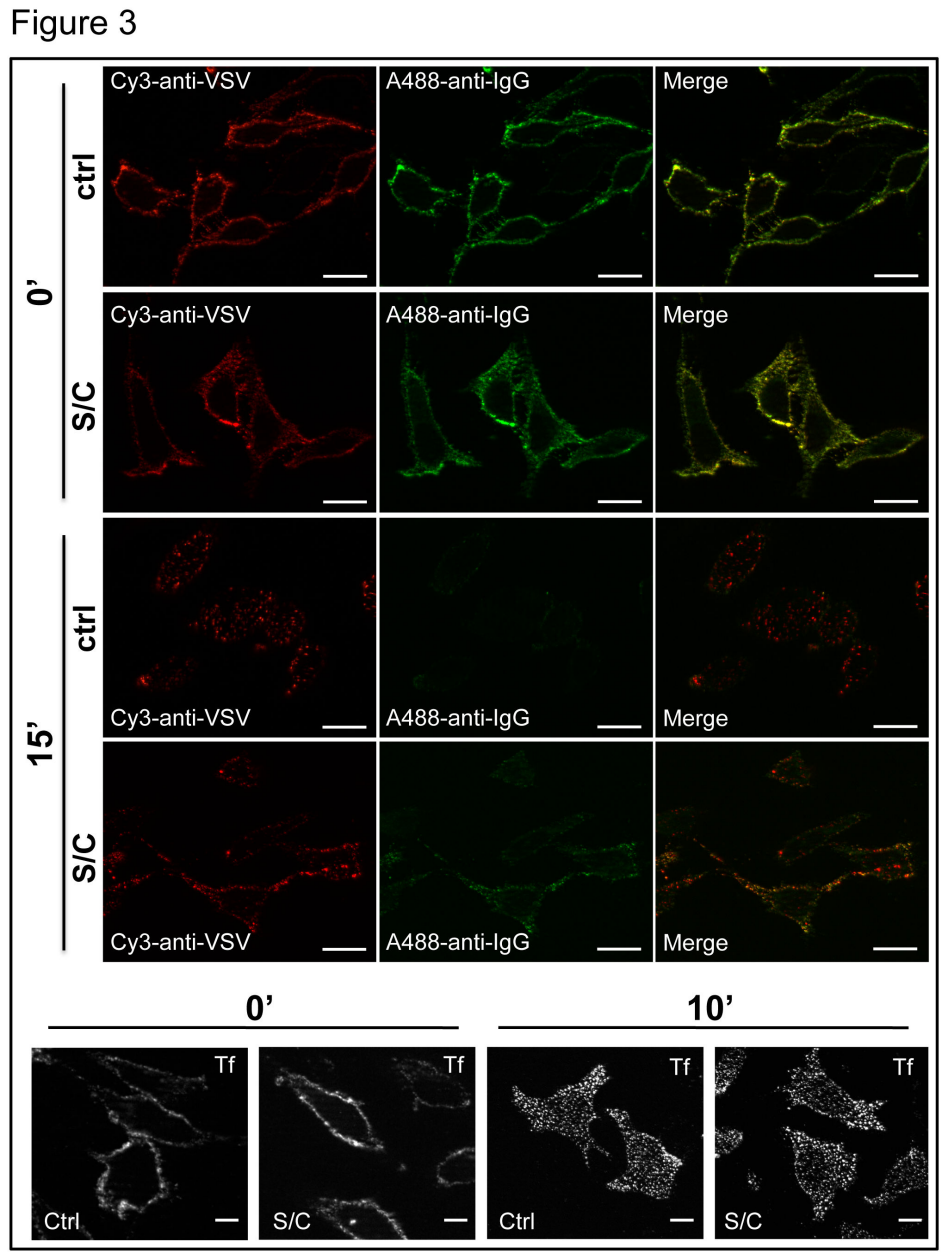
Cell treatment with smase/coase interferes with Dll1 internalization. HeLa cells stably transfected with VSV-Dll1 were serum-starved for two hours before being treated (S/C) or not (control) with smase and coase for 1 hour. They were then incubated on ice with a Cy3-coupled anti-VSV antibody, and returned to 37°C in the presence (S/C) or absence (ctrl) of smase and coase for 15 minutes (or fixed immediately after the incubation with the anti-VSV, panels 0'), fixed, washed and incubated with Alexa 488-coupled anti-IgG antibody (to label Dll1 present at the cell surface, which had already been marked with anti-VSV), before being processed for immunofluorescence. Labeling with coupled anti-IgG alone showed no staining (not shown). Using Tf-Cy3, the internalization of transferrin (Tf) was analyzed in the same conditions. Scale bar, 10 µm.

We then tested the consequence of smase/coase treatment on Dll1 trafficking. The internalization of VSV-tagged Dll1, transiently-transfected into HeLa cells, was monitored by dye-coupled antibody uptake experiments, followed by staining of the Dll1 molecules which are still present at the plasma membrane (see Materials and Methods). Figure 3 shows that smase/coase treatment interfered with Dll1 internalization; after 15 minutes of antibody uptake, surface staining of Dll1 was no longer visible in control cells while it was still clearly visible in smase/coase treated cells. On the other hand the uptake of transferrin, known to be essentially endocytosed through clathrin-mediated endocytosis, was not modified when cells were treated with these drugs (lower panel). Taken together these results indicate that correct internalization of Dll1 requires its association with certain components of the plasma membrane such as cholesterol and/or sphingomyelin, and possibly with membrane subdomains enriched in these components.

### The glycosphingolipid-binding motif of Dll1 is implicated in ligand trafficking and stability

**Figure 4:**
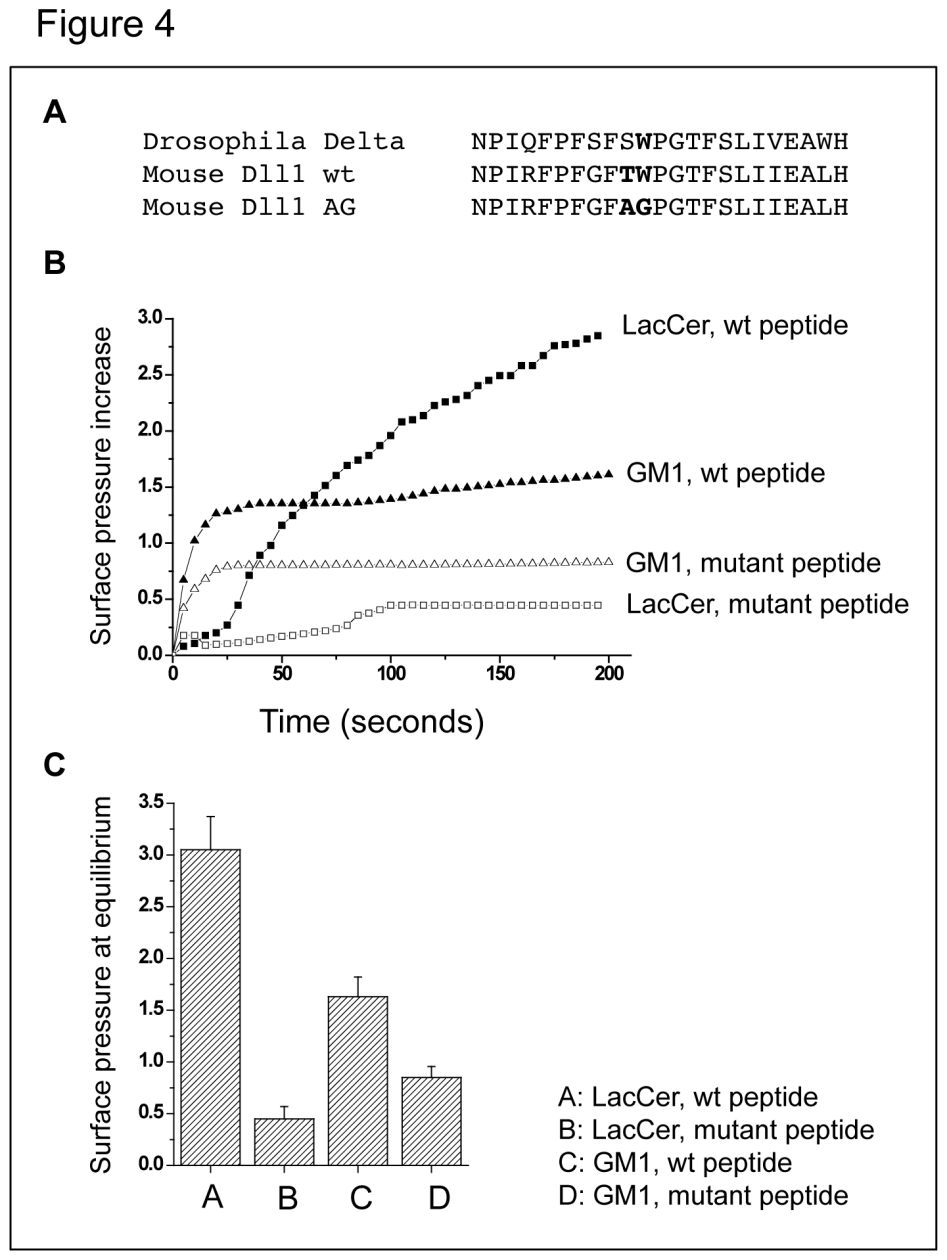
The putative GBM of Dll1 binds glycosphingolipids and requires the central Trp residue. (A) Amino acid sequence alignment of Drosophila Delta and mouse Dl11 covering the Drosophila GBM. The sequence shown for the murine Dll1 wt and Dll1 AG corresponds to the synthetic peptides used in panels B and C. (B and C) Interaction of the synthetic peptides Dll1 wt and Dll1 AG (mutant peptide) with LacCer and GM1 ganglioside monolayers was measured using the Langmuir film balance technique. The binding kinetics are shown in B, and in C the increase in maximal surface pressure (expressed in mN/m) elicited by the peptides was determined after equilibrium has been reached. Error bars indicate standard variation (3 determinations).

**Figure 5:**
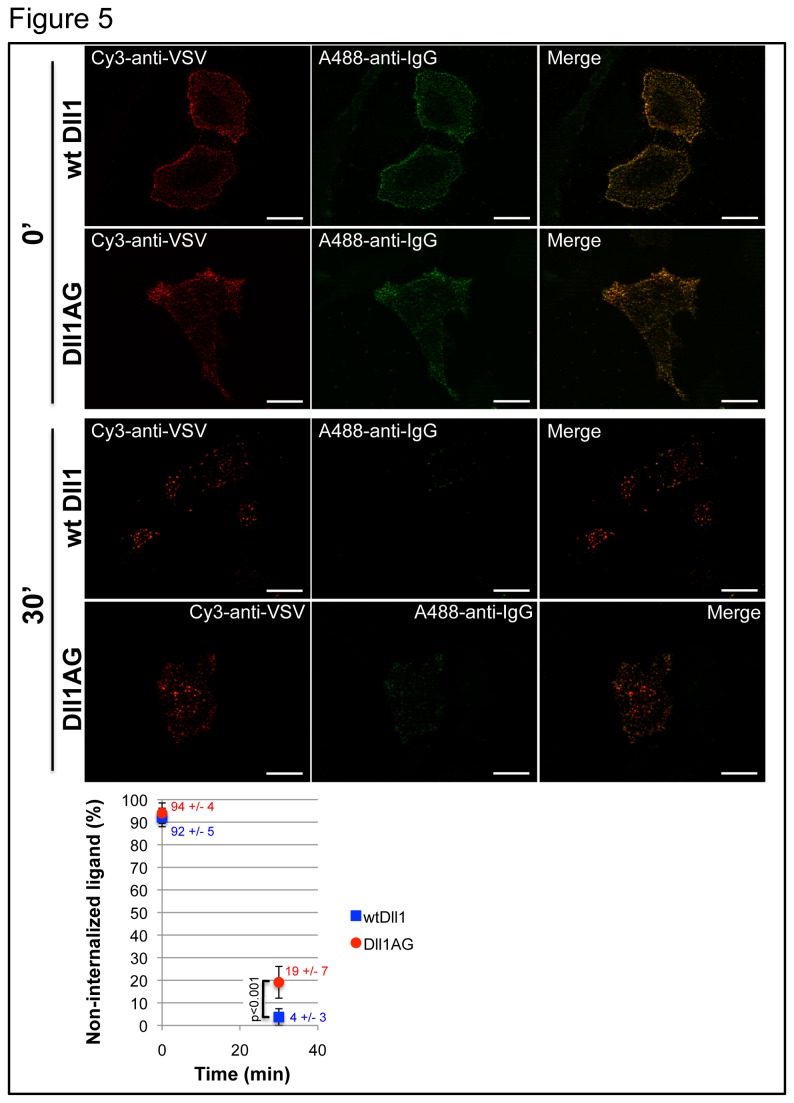
Internalization of Dll1AG. Hela cells were transiently-transfected with VSV-Dll1 or VSV-Dll1AG. Cy3-coupled anti-VSV antibody uptake (for 0 or 30 minutes), and secondary labeling with Alexa 488-coupled anti-IgG (to label Dll1 present at the cell surface) were performed as described in the legend to Figure 3. The presented image is typical of the results obtained in multiple experiments. The graph at the bottom of the figure presents the quantitation of the abovementioned experiment, in which the proportion of wt or mutant ligand remaining at the cell surface is plotted against time. For each condition, 20 images were quantified. Values on the graph are indicated +/- standard deviation. The p-value was calculated using MATLAB. Scale bar, 10 µm.

We then determined whether interfering with Dll1 association with certain plasma membrane components ultimately interferes with the ability of Dll1 to signal to the Notch receptor. *Ex vivo* measurement of Notch activation usually relies on an assay involving coculture of Notch-expressing cells (transfected with a Notch reporter gene), and of ligand-expressing cells [37]. However to evaluate the importance of membrane composition, we could not use smase/coase treatment as it would affect both signal-sending and -receiving cells in our coculture assay. As glycosphingolipids are known to interact with sphingomyelin [38], and are thus likely to colocalize in the same membrane subdomains, we explored the impact of a putative motif (GBM) known to trigger interaction with glycosphingolipids, identified in the extracellular domain of Drosophila Delta. The GBM is apparently conserved in vertebrate homologues including mouse Dll1 ( [28], Figure 4A: residues 105-127). This motif consists of a hairpin structure containing a solvent-exposed aromatic residue (Trp, in position 115 in the case of murine Dll1) which plays a prominent role in protein-sugar interaction [39]. The functionality of this putative motif was demonstrated by analyzing, *in vitro*, the interaction between a synthetic peptide corresponding to this motif (mouse Dll1 wt in Figure 4A) and purified glycosphingolipids using the Langmuir film balance technique [39]. The neutral glycosphingolipid LacCer and the ganglioside GM1 were prepared as a monolayer at the air-water interface, and the synthetic Dll1 peptide was injected in the aqueous phase. The intensity of the interaction was measured by the increase with time of the surface pressure of the film. Results presented in Figure 4 (B and C) show that the wt peptide interacts strongly with LacCer and more weakly with GM1. These interactions were disrupted when the critical Trp residue in the GBM was mutated to glycine (mouse Dll1 AG peptide in panel A). These experiments confirm the existence of a GBM in the extracellular part of murine Dll1, and the importance of Trp115 for the interaction between the GBM and glycosphingolipids. To gain insight into the role of this motif in the regulation of Dll1 trafficking and signaling activity, we introduced the Trp115Gly mutation in the context of the full-length molecule: the resulting ligand was called Dll1AG. Because the data in figures 2 and 3 demonstrate the importance of Dll1 association with certain lipid components of the plasma membrane for its trafficking and stability, we investigated the possibility that the mutation of the GBM affects the trafficking and ultimately the signaling activity of Dll1AG. To test this hypothesis, dye-coupled antibody uptake and secondary labeling of Dll1 wt or Dll1AG localized at the cell surface (similar to the procedure used in Figure 3) were performed in HeLa cells, transiently-transfected with VSV-Dll1 or VSV-Dll1AG. The percentage of Dll1 which was not internalized was measured at 0 and 30 minutes of internalization (see Materials and Methods) and plotted at the bottom of Figure 5. At time 0, as expected, almost 100% of wt Dll1 and Dll1AG were expressed at the cell surface (Figure 5). After a 30 min chase at 37°C, 4 +/- 3% of wt Dll1 were present at the cell surface, while 19 +/- 7% of Dll1AG were still present at the cell surface. This significant difference was reproducible in several experiments. These results suggest that mutating the GBM interferes with proper Dll1 endocytosis.

In order to study in more details the consequences of the GBM mutation on Notch signaling, we first tested the ability of Dll1AG to recycle, a property which we previously demonstrated to be required for Dll1 activity [26]. We then measured the turnover of Dll1 AG and finally its ability to activate the pathway in a cell-coculture assay.

**Figure 6:**
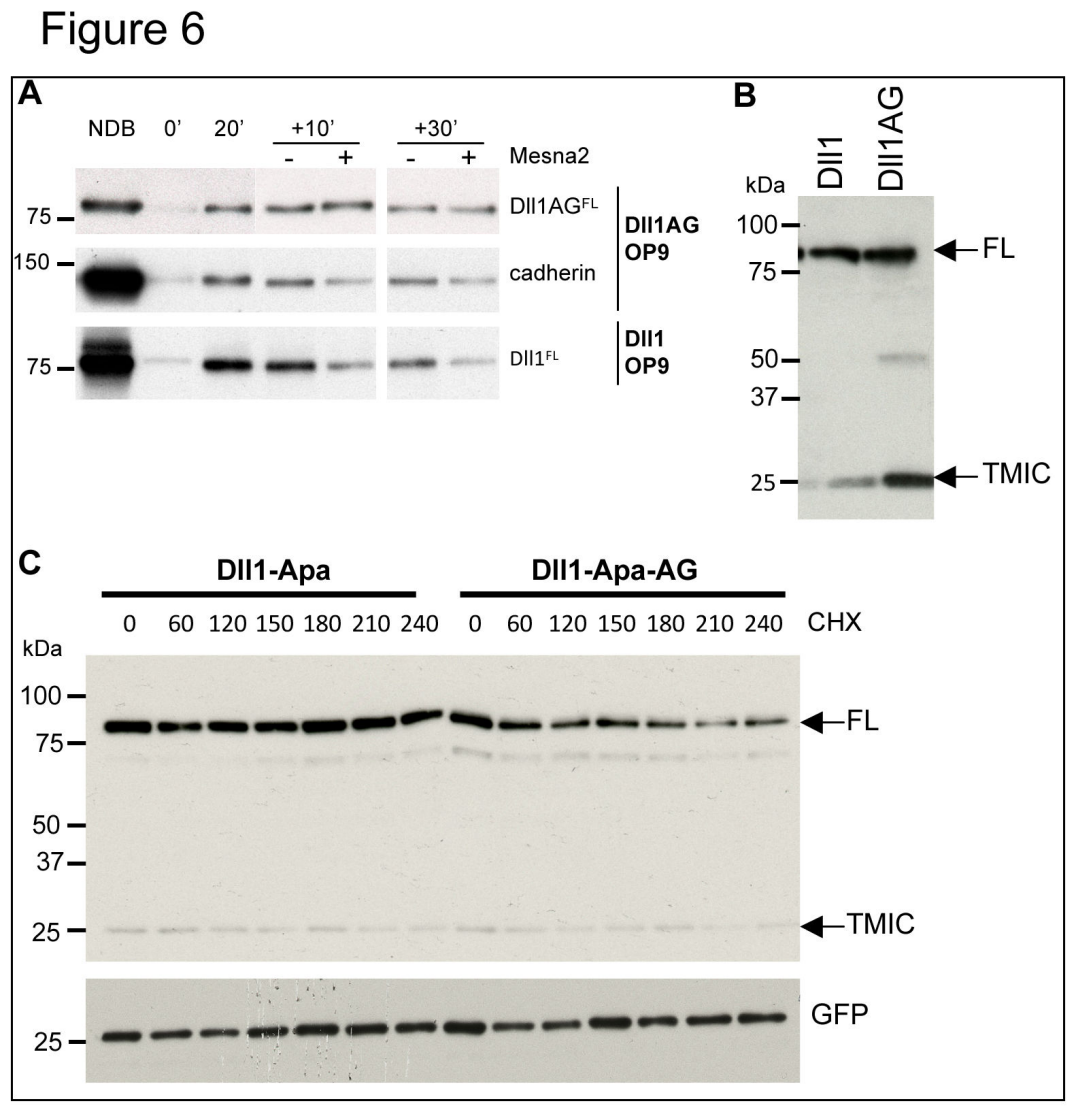
Dll1AG does not recycle and exhibits a shorter half-life than wild type Dll1. (A) Surface proteins of Dll1- and Dll1AG-expressing OP9 cells were labeled with biotin and a recycling assay was performed as described in Materials and Methods. NDB: not debiotinylated; Mesna2: second Mesna treatment. (B) Western blot analysis of cells expressing Dll1 or Dll1AG. Approximate equal loading of the full-length form was used in order to facilitate estimation of the extent of metalloprotease cleavage. (C) HeLa cells were transiently-transfected with plasmids encoding Dll1-Apa or Dll1-Apa-AG together with GFP. Following cycloheximide treatment the levels of ligands were monitored as described in Figure 2. Western blots were performed using anti-Dll1 and anti-GFP antibodies.

Recycling was tested using a reversible biotinylation assay described in Heuss et al (2008). After cell surface biotinylation and 20 minutes of endocytosis at 37°C, a first MesNa treatment aimed at removing any accessible biotin at the cell surface (Figures 6A, 20’), showed that wt Dll1 and Dll1AG had been internalized. Cells were then incubated at 37°C to allow transport through recycling endosomes for various periods of time (10 or 30 min). At each time point, some cells were reexposed to MesNa to strip biotin from ligands that had recycled the cell surface (Figure 6A, Mesna2). In contrast to Dll1, the level of biotinylated Dll1AG mutant remained identical irrespective of the second MesNa treatment, indicating that it is unable to recycle. As an internal control, we used endogenous cadherin which, contrary to Dll1AG, exhibits efficient recycling.

To assess whether the inability of Dll1AG to recycle is associated with trafficking along a degradative pathway, we measured the half-life of Dll1 and Dll1AG in cycloheximide-treated cells. Experiments using wt Dll1 and Dll1AG (Figure 6B) indicate that metalloprotease mediated processing of Dll1AG, which likely occurs at the cell surface, was enhanced compared to the wild type ligand (Figure 6B), similar to the situation observed in smase/coase-treated cells (see Figure 1C). To accurately explore the turnover of full-length Dll1AG, we generated a non-cleavable ligand Dll1-Apa-AG (modeled on the Dll1-Apa described in Figure 2) and monitored its turnover. As shown in Figure 6C, this mutant was rapidly degraded following cycloheximide treatment. All together these results are reminiscent of those obtained after smase/coase treatment of cells expressing the wild type ligand, and suggest that the GBM motif of Dll1 is required for the ligand to be internalized and escape degradation and shedding.

### Dll1AG is unable to activate Notch signaling

**Figure 7:**
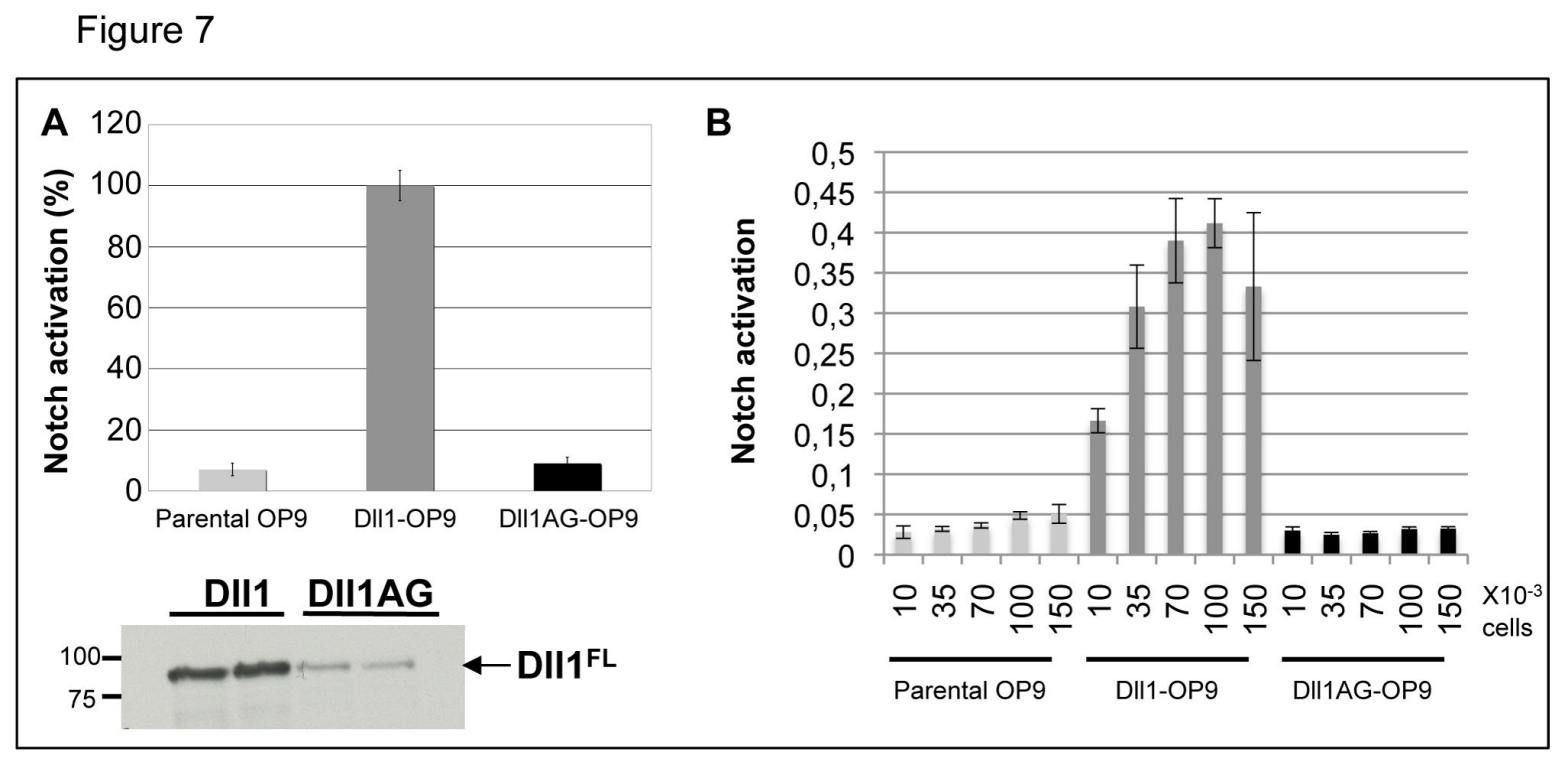
Dll1AG does not activate Notch signaling. U2OS cells, stably expressing HA-tagged Notch1, were transiently-transfected with a CSL-luciferase construct and pRL-TK-renilla luciferase, as described in Materials and Methods. (A) Twenty-four hours after transfection, 7x10^4^ OP9 cells stably expressing either VSV-Dll1 (dark gray bars, Dll1-OP9), VSV-Dll1AG (black bars, Dll1AG-OP9) or control cells (light gray bars, parental OP9) were added. Luciferase activity was measured after 20 hours of coculture. The relative luciferase activity in the presence of Dll1-OP9 was defined as 100%. Bottom panel shows the expression levels of full-length Dll1 and Dll1AG in the cell lines used for coculture. (B) Increasing amounts of Dll1-OP9 and Dll1AG-OP9 cells were cocultured with Notch1 expressing cells and the Notch reporter activity was measured. Error bars represent the standard variation of triplicate experiments.

To test whether Dll1AG is able to activate Notch signaling, we performed a coculture assay of U2OS cells stably expressing HA-tagged Notch1 (N1HA-U2OS), transiently-transfected with a Notch-dependent luciferase reporter gene (CSL-firefly luciferase), with OP9 cells stably expressing wt Dll1 or Dll1AG (Figure 7). The relative luciferase activity was determined by normalizing CSL-firefly luciferase with control renilla luciferase. In parallel cell extracts were analyzed by Western blot: quantification of the blot shown in panel A indicates that the amount of full-length Dll1AG is four time lower than that of the wild type ligand. This difference is likely due to differential cleavage of Dll1 and Dll1AG and to the shorter half-life of the mutant. As a consequence, despite the use of conditions where the total amount of Dll1 and Dll1AG are identical, the final amount of full-length molecules may vary from one experiment to the other and is hard to predict. In order to minimize this bias, we tested Notch activation in the presence of increasing amounts of Dll1- or Dll1AG-expresssing cells, over a range of 15 fold. The results indicate that Dll1AG is not able to activate Notch signaling (Figure 7B), irrespective of the amount of ligand-expressing cells added to the receptor-expressing cells. While reporter expression was stimulated when only 10,000 Dll1- expressing cells were cultured with N1HA-U2OS, no stimulation was observed with 150,000 Dll1AG-expressing cells (Figure 7B). These results show that the ability to interact with glycosphingolipids is primordial for DSL ligand activity.

### Silencing glucosylceramide synthase by shRNA attenuates Notch activation

**Figure 8:**
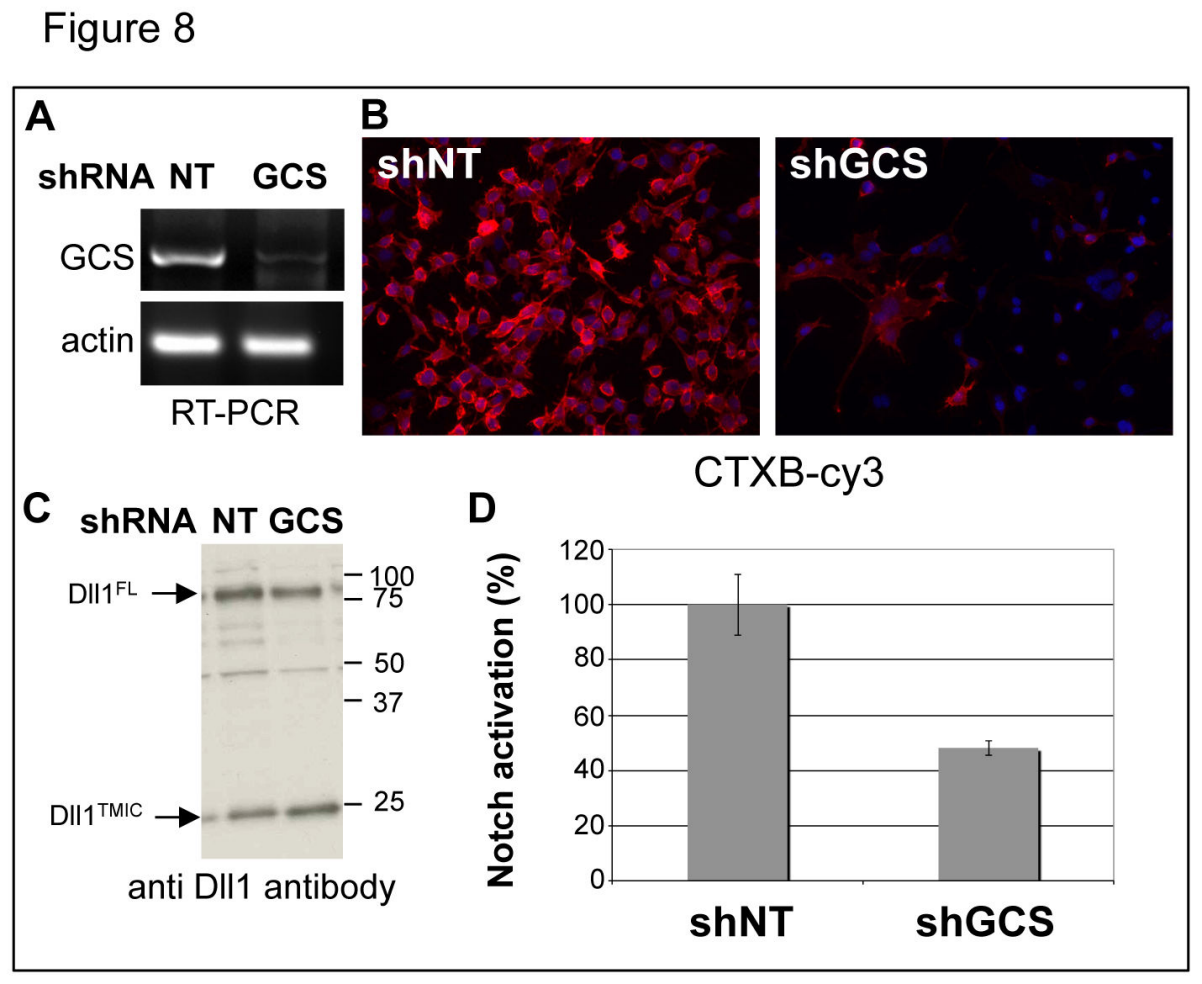
Down-regulation of glucosylceramide synthase (GCS) affects Notch activation. (A) After lentivirus-mediated transduction with shRNA targeting GCS (GCS) or control shRNA (NT), MEFs stably expressing VSV-Dll1 were selected with puromycin before being assayed for actin and GCS mRNA by semi-quantitative RT-PCR. (B) Cells stably transduced with the GCS or the control shRNA were surface labeled on ice with CTXB-Cy3 prior to fixation. Images were acquired using a 20x objective. Scale bar 20 µm. (C) Whole cell extracts of cells transduced with the GCS or the control shRNA were analyzed by Western blot using Dll1 antibody. (D) The impact of GCS silencing on Notch activation was tested in a coculture assay. Cells described in panel A were cocultured with a U2OS line expressing the Notch1 receptor. Notch activation was evaluated using a CSL reporter strategy as described in Figure 7. Error bars represent standard variation. The presented result is representative of four independent experiments.

To confirm that glycosphingolipids are required for proper activity of Dll1, we decided to down-regulate glucosylceramide synthase (GCS) using RNA interference in MEFs stably expressing VSV-Dll1. GCS catalyzes the first step in ganglioside synthesis by transferring glucose residues of UDP-glucose onto ceramide to produce glucosylceramide. Stable expression of an shRNA targeting GCS was generated by lentiviral transduction. Semi quantitative RT-PCR showed that GCS shRNA significantly reduced GCS mRNA (Figure 8A), compared to non-targeting shRNA (NT). To prove that expression of the shRNA interfered with the synthesis of gangliosides, we monitored the binding to the cell surface of cholera toxin subunit B (CTXB), which is known to be internalized through interaction with gangliosides [30]. As shown in Figure 8B, CTXB bound efficiently to the surface of MEFs expressing non-targeting shRNA (NT) but failed to bind to cells transduced with the GCS shRNA. Western blot analysis of Dll1 (Figure 8C) indicated that reducing the level of expression of GCS did not significantly affect the total amount of Dll1. To test whether down-regulation of gangliosides in signal-sending cells could act on Notch signaling, we performed a coculture assay in serum-free medium (to avoid the presence of exogenous gangliosides). Cells transduced with the GCS shRNA showed a 50% reduction of Notch activity compared to cells transduced with control shRNA (NT) (Figure 8D). These results confirm the importance of glycosphingolipids in the activity of the Dll1 ligand.

## Discussion

Understanding the mechanisms that control Dll1 endocytosis is crucial because ligand internalization in signal-sending cells determines the intensity and timing of the signaling activity. Endocytosis has been suggested to be required either during a so-called "activation" trafficking event of the ligand which takes place before contact with the Notch receptor, or during the transendocytosis event that occurs following contact of the ligand with the receptor and results in a structural change in Notch, allowing cleavage by a metalloprotease and subsequent activation of the signaling cascade. We favor a model where these 2 events would take place consecutively, and we focus here on the "activation"-linked trafficking events, that occur independently of the presence of the receptor. This has important consequences: a number of the published studies focus on the transendocytosis event, or are performed in conditions where it is difficult to determine whether the conclusions concern the first or the second trafficking event mentioned above. In this study, a number of experiments deal with trafficking of the ligand *per se* and are independent of the presence of the receptor.

A number of endocytosis pathways have been described so far [20]. Interestingly the existence of two alternative routes of internalization has been demonstrated for certain cell surface receptors (for example EGF receptor, TGFß receptor and LRP6), leading to either activation or inhibition of the cognate signaling pathways [40]. In this manuscript we show that the Notch ligand Dll1 interacts with lipid components of the plasma membrane through a specific motif located in its extracellular domain, and that this interaction is necessary for proper endocytosis and protection from shedding and degradation.

Because we have previously shown that the ligand Dll1 can be found in DRMs following detergent treatment and sucrose gradient, and that inactive mutants of Dll1 do not localize to these fractions [26], we investigated in this study the mechanisms by which Dll1 associates with specific domains of the plasma membrane. For this we followed the intracellular trafficking and fate of Dll1 under conditions where membrane lipid organization has been disturbed. We then studied the characteristics of Dll1 mutated on a putative glycosphingolipid-binding domain and confirmed that Dll1 trafficking and activity can be regulated by its lipid environment.

### Lipid organization of the plasma membrane determines proper trafficking of Dll1 and protection from shedding and degradation.

Shedding by cell surface metalloproteases ADAMs [18] or MT1-MMP [41] represents another mode of regulation of Dll1 activity. We have observed that disturbing plasma membrane organization through the use of sphingomyelinase and cholesterol oxidase (smase/coase) increases Dll1 shedding and reduces its stability, similar to the situation observed with Dll1AG, a mutant molecule unable to interact with glycosphingolipids. Shedding of membrane proteins like CD30, p75, IL-6 and APP were also shown to be dependent on plasma membrane subdomains [33,42,43]. These findings can be explained if interfering with the organization of these subdomains inhibits endocytosis of these proteins and increases their concentration at the cell surface, where they can undergo metalloprotease cleavage. Indeed the majority of ADAM10, the major protease responsible for shedding of Notch ligands, is present at the cell surface (and is excluded from lipid rafts) [44,45]. In this case, constitutive endocytosis would permit storage and recycling of full-length active ligands. Alternatively, certain lipid components of the membrane (such as glycosphingolipids, see below) may protect the ligands from metalloprotease cleavage.

The decreased stability of Dll1 (as measured using a shedding-resistant Dll1 mutant) following either smase/coase treatment or mutation of its glycosphingolipid-binding motif can be explained if the subsequent perturbation of Dll1 microenvironment results in its targeting to a degradative pathway (such as lysosomes).

### Glycosphingolipids play a critical role in Dll1 signaling activity

Recent results [28] describing a glycosphingolipid-binding motif (GBM) in the extracellular region of Drosophila Delta led us to look for a similar sequence in murine Dll1. Common GBM’s consist of a hairpin structure containing a water-exposed aromatic residue [39]. Using the Langmuir monolayer technique, we identified a GBM in the extracellular domain of mouse Dll1, and showed that mutating its conserved aromatic residue (Trp) abolished interaction with glycosphingolipids. We also demonstrated that mutation of this critical Trp residue increases Dll1 shedding, reduces its half-life and prevents its recycling following endocytosis, eventually abolishing its ability to activate Notch in a coculture assay. Strikingly, the behavior of the AG mutant of Dll1 is highly reminiscent of that of wt Dll1 in smase/coase-treated cells.

Glucosylceramide synthase (GCS) is a key enzyme for glycosphingolipid synthesis. Suppressing GCS in signal-sending cells with shRNA reduced the level of glycosphingolipids, and down-regulated Notch activation following coculture. All together these results indicate that glycosphingolipids play an important role in the function of Dll1.

Several non-mutually exclusive interpretations of these data can be proposed. Interaction of the ligand with glycosphingolipids may be essential to prevent its entry into degradative compartments. The loss of interaction with glycosphingolipids might also cause the mislocalization of ligands to a different subclass of membrane microdomains that cannot promote ligand signaling activity. Another possibility is that glycosphingolipids can induce a conformational change or clustering of the ligands, which increase their affinity for the Notch receptor.

In conclusion, the current study presents evidence that lipid organization of the plasma membrane and interaction with glycosphingolipids play a critical role in the selection of the internalization route of Dll1, which regulates its stability and signaling activity.

## Materials and Methods

### DNA constructs

A pcDNA3-based plasmid expressing VSV-tagged Dll1AG (vsvDll1AG-pcDNA3) was obtained by site-directed mutagenesis of VSV-Dll1-pcDNA3 [46] using the oligonucleotide 5’-ATCCGATTCCCCTTCGGCTTCGCCGGCCCAGGTACCTTCTCTCTGATC-3’ and its complementary DNA to convert the threonine 114 and the tryptophan 115 of wild type Dll1 into an alanine and a glycine respectively, and to create a NaeI site. Dll1-Apa construct was described in [36]. Dll1-Apa-AG was derived from this construct by site-directed mutagenesis. CSL-firefly luciferase was a gift from T. Honjo (Kyoto University, Japan).

### Cells, transfections, antibodies and chemicals

HeLa, OP9 and U2OS cell lines, and SV40-transformed mouse embryo fibroblasts (MEF) have been obtained from ATCC. HeLa cells were transiently-transfected with FuGene 6 (Roche) and processed for immunofluorescence after 24 hours. Stable Dll1-expressing OP9 cells and MEFs have been obtained by retroviral transduction and described in [46] and [36], respectively. HeLa cells stably expressing Dll1 were obtained by lentiviral transduction, followed by puromycin selection. The U2OS cell line stably expressing HA-tagged Notch1 has been described in [37]. Whole cell extracts and immunoblots were carried out as previously described [46].

The following antibodies were used (WB: Western Blot, IF: immunofluorescence): rabbit anti-Dll1 [36] (WB 1/5000), anti-EGF receptor (WB 1/4000, Santa Cruz), anti transferrin receptor (WB 1/1000, Invitrogen), monoclonal Cy3-coupled anti-VSV (1/1000, Sigma), Transferrin (Tf) conjugated to Cy3 was a gift from N. Sauvonnet. Cholera Toxin B subunit Cy3 conjugate was purchased from Sigma.

Cells were first serum-starved for 2 hours before treatment with smase and coase for 1 hour. Sphingomyelinase (smase, Sigma) and cholesterol oxidase (coase, Calbiochem) were used at 0.1 unit/mL and 1 unit/mL respectively.

### DRM preparation

DRMs were isolated from cells lysed in 1% Brij 98 and prepared as described in [26].

### Recycling assay using reversible biotinylation

A detailed description of this assay has been published previously [26]. Stably-expressing OP9 cells were incubated on ice with cleavable biotin (NHS-SS-biotin, Pierce) then shifted 20 min to 37°C and underwent a first reducing treatment with MesNa. Then cells were incubated again at 37°C, treated or not with MesNa, lysed and biotinylated proteins isolated on streptavidin-agarose were analyzed by immunoblot.

### Immunofluorescence Assays

For antibody uptake experiments, cells stably or transiently-expressing Dll1 were incubated on ice with Cy3-coupled anti-VSV antibody. They were then incubated for various periods of time at 37°C, fixed with 4% paraformaldehyde, washed and incubated with Alexa 488-coupled anti-IgG secondary antibody (Life Technologies), which only stains the Dll1 molecule which are present at the cell membrane (and have been marked with the anti-VSV antibody). After washing cells were mounted in Mowiol. Images were acquired using AxioImager Z1 microscope, using the 63x objective, with 0.3 µm sections using AxioVision Rel. 4.8 with Apotome system (Carl Zeiss MicroImaging Inc.). Quantification was performed using the JACoP plugin of ImageJ [47]. Manders’ coefficient for the Cy3 channel was calculated using the plugin to determine the proportion of Cy3 signal colocalizing with Alexa 488 signal, and thus to plot the proportion of wt or mutant ligand still remaining at the cell surface against time. For the statistical analysis, the data were analyzed using MATLAB (The Mathworks, Natick, USA).

MEFs stably expressing Dll1 and a shRNA targeting glucosylceramide synthase (GCS) (or a control vector) were incubated with 1μg/mL CTXB-Cy3 on ice, washed four times and fixed. Immunofluorescence analysis was performed as described [46].

### Measurement of ligand half-life

Cells were transfected and then incubated at 37°C with 50 µg/mL cycloheximide (Sigma) for various periods. Cells were lysed with 1% NP-40 and ligand levels were analyzed by Western Blot. Quantity One software (Biorad) was used to assess the amount of Dll1. GFP was used as a loading control.

### Peptide-glycosphingolipid interaction

Synthetic peptides (purity 95%) were purchased from Schafer-N (Copenhagen). Surface pressure measurements revealing peptide-lipid interactions were studied by the Langmuir film balance technique with a fully automated microtensiometer as described previously [39]. Peptides (10 mM) were injected under a monomolecular film of the indicated purified glycosphingolipids and the pressure increases were recorded kinetically until the equilibrium was reached.

### Dual-luciferase assay

4 x 10^4^ HANotch1-U2OS cells were plated in 24-well plates. 24 hours after, cells were transfected with 0.25 µg of CSL-luciferase and 0.25 µg of renilla luciferase expressing plasmid (pRL-TK, Promega). 24 hours after transfection, various amount of cells expressing ligand were added to HANotch1-U2OS cells. 18 hours later, cocultures were lysed using Passive lysis buffer (Promega). Firefly luciferase and renilla luciferase activity were measured using the luminometer Centro XS (Berthold). Relative luciferase activity was determined by normalizing CSL-firefly luciferase with renilla luciferase.

### Transduction of shRNA/GCS into MEF-Dll1 cells

Short hairpin (sh) RNA lentiviral particles based on pLko.1, targeting the mouse GCS was purchased from Sigma Mission RNAi (NM_011673) and used to transduce MEF cells expressing Dll1 at multiplicity of infection to 3. Cells were then selected by puromycin (2 µg/mL). As a control cells were transduced with a viral vector containing non coding shRNA (Sigma).

### RT-PCR

Total RNA was isolated using the RNeasy Mini Kit (Qiagen). Reverse transcription was performed using 200 units Superscript II Reverse Transcriptase (Invitrogen), 2 μg of total RNA and 600 ng of random primers. RT-PCR was performed using Taq DNA polymerase (Taqara). The forward primer was ACGGGCTGCCGTATGTAGCCG and the reverse primer CTGAATACATGGTGGGCTGCCC. The amplified fragment analyzed by agarose gel electrophoresis was 600 bp. Actin was used as internal standard. Amplification was analyzed after 25 to 35 rounds of cDNA synthesis. Exponential amplification of GCS occurs during cycle 27-30.
